# In Vitro ADME Properties of Two Novel Antimicrobial Peptoid-Based Compounds as Potential Agents against Canine Pyoderma

**DOI:** 10.3390/molecules23030630

**Published:** 2018-03-10

**Authors:** Ines Greco, Bernard D. Hummel, Jaspreet Vasir, Jeffrey L. Watts, Jason Koch, Johannes E. Hansen, Hanne Mørck Nielsen, Peter Damborg, Paul R. Hansen

**Affiliations:** 1 Department of Drug Design and Pharmacology, University of Copenhagen, Universitetsparken 2, 2100 Copenhagen Ø, Denmark; rwn546@alumni.ku.dk; 2 Department of Food Science, University of Copenhagen, Rolighedsvej 30, DK-1958 Frederiksberg, Denmark; 3 Zoetis Inc., 333 Portage St., Kalamazoo, MI 49007, USA; bernard.d.hummel@zoetis.com (B.D.H.); jaspreet.vasir@zoetis.com (J.V.); jeffrey.l.watts@zoetis.com (J.L.W.); jason.koch2@zoetis.com; (J.K.); 4 Department of Pharmacy, University of Copenhagen, Universitetsparken 2, 2100 Copenhagen Ø, Denmark; hanne.morck@sund.ku.dk; 5 Department of Veterinary and Animal Sciences, University of Copenhagen, Stigbøjlen 4, 1870 Frederiksberg C, Denmark; pedam@sund.ku.dk

**Keywords:** antimicrobial peptoids, peptidomimetics, in vitro ADME, topical formulation, metabolic stability

## Abstract

Antimicrobial peptides (AMPs) hold promise as the next generation of antimicrobial agents, but often suffer from rapid degradation in vivo. Modifying AMPs with non-proteinogenic residues such as peptoids (oligomers of *N*-alkylglycines) provides the potential to improve stability. We have identified two novel peptoid-based compounds, **B1** and **D2**, which are effective against the canine skin pathogen *Staphylococcus pseudintermedius*, the main cause of antibiotic use in companion animals. We report on their potential to treat infections topically by characterizing their release from formulation and in vitro ADME properties. In vitro ADME assays included skin penetration profiles, stability to proteases and liver microsomes, and plasma protein binding. Both **B1** and **D2** were resistant to proteases and >98% bound to plasma proteins. While half-lives in liver microsomes for both were >2 h, peptoid **D2** showed higher stability to plasma proteases than the peptide-peptoid hybrid B1 (>2 versus 0.5 h). Both compounds were suitable for administration in an oil-in-water cream formulation (50% release in 8 h), and displayed no skin permeation, in the absence or presence of skin permeability modifiers. Our results indicate that these peptoid-based drugs may be suitable as antimicrobials for local treatment of canine superficial pyoderma and that they can overcome the inherent limitations of stability encountered in peptides.

## 1. Introduction

**Figure 1:**
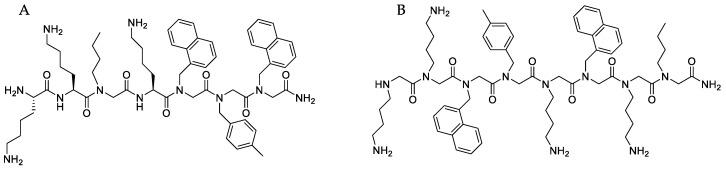
Structure of **B1** (**A**) and **D2** (**B**) both isolated as trifluoacetic acid salts.

The emergence of bacterial pathogens resistant to multiple antimicrobial agents over the past decades is a growing public health threat, and there is an urgent need for novel antimicrobial drugs for human and veterinary use [1,2]. In companion animals, canine skin infections constitute the main cause of antibiotic use [3]. *Staphylococcus pseudintermedius* is the primary pathogen of this disease, as it is isolated from around 90% of canine clinical skin samples [4]. In the recent decade, the worldwide emergence of methicillin-resistant *S. pseudintermedius* (MRSP) [5] has resulted in infections untreatable with antimicrobial agents licensed for systemic use in dogs. Therefore, there is an urgent need to develop alternative antimicrobial agents to combat this pathogen. Furthermore, systemic administration of antibiotics may promote the development of resistance and current guidelines encourage the use of topical therapy for treatment of MRSP infections [6]. Peptides with antimicrobial activity (AMPs) are the object of rapidly-growing research interest as the next generation of antibacterials [7,8,9]. In general, AMPs are small (<50 amino acids), cationic, amphipathic host defense peptides naturally present in the innate immune systems [10]. AMPs generally show specificity for bacterial targets, broad-spectrum activity and lower affinity for mammalian host cells [11,12,13,14]. Although promising, they display limited stability in vivo being easily degraded by proteases [15]. In recent years, chemical and structural modifications have been used as strategies for improving stability of peptides. Structural analogues of peptides such as peptoids (polymers of *N*-substituted alkylglycines) [16,17] have been introduced to overcome stability issues. Introduction of peptoid residues in the structure of existing or de novo designed AMPs has also become a successful approach to improve their activity and pharmacokinetic properties [15]. Whereas effort has been made to assess the structure-activity relationship of antimicrobial peptoids to improve activity [18], their ADME (absorption, distribution, metabolism and excretion) profile has not been investigated to the same extent. In the present study, these aspects were characterized for a peptide-peptoid hybrid (**B1**) and a peptoid (**D2**) designed as antimicrobials for topical use against *S. pseudintermedius*. The structures of **B1** and **D2** are shown in Figure 1. First generation formulations of **B1** and **D2** for topical administration have been developed and used for testing. The in vitro ADME profile of **B1** and **D2** in terms of degradation by metabolic enzymes, plasma protein binding and permeation through excised dog skin were investigated in this study.

## 2. Results

### 2.1. MIC and Hemolytic Activity In Vitro

**Figure 2:**
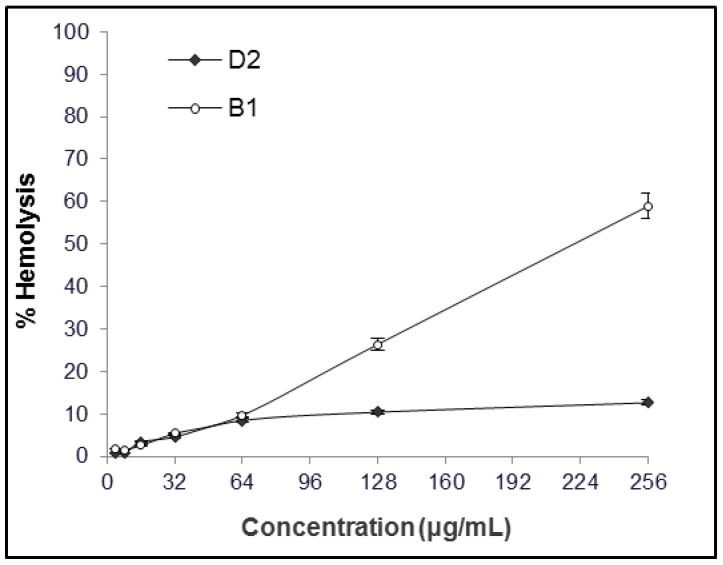
Hemolytic activity of peptide-peptoid hybrid **B1** and peptoid **D2** in human erythrocytes as a function of concentration. Mean ± SEM, *n* = 3.

**B1** and **D2** were selected after screening of a combinatorial library of de novo designed compounds for their activity against *S. pseudintermedius* and host toxicity in vitro measured as lytic activity against red blood cells. **B1** and **D2** were designed on the basis that the majority of antimicrobial peptides are cationic and hydrophobic. We envisaged that short linear peptide-peptoid hybrids consisting of cationic lysines and hydrophobic peptoid residues would result in derivatives with potent antibacterial activity and low cytotoxicity. Minimum inhibitory concentrations against *S. pseudintermedius* were 2–4 μg/mL for **B1** and 1–2 μg/mL for **D2**, irrespective of the strain being MSSP or MRSP. Preliminary data suggest that **B1** and **D2** also are active against a wide range of canine pathogens (data not shown, Greco et al., manuscript in preparation). The hemolytic activity of **B1** and **D2** as a function of their concentration is shown in Figure 2. Repeated experiments on different days with different human blood samples confirmed the results. The concentration of compound resulting in 10 and 50% hemolysis of erythrocytes (EC_10_ and EC_50_) were 64 µg/mL and 219 μg/mL for **B1** and 128 µg/mL and >256 μg/mL for **D2**. In a separate experiment, performing the test with dog blood resulted in hemolysis at 150 μM of 45% and 22% for **B1** and **D2**, respectively.

### 2.2. Stability to Proteases

**Figure 3:**
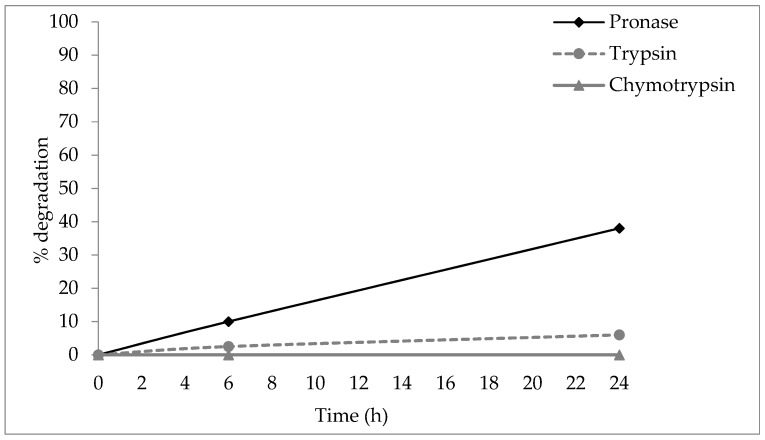
Proteolytic degradation of peptide-peptoid hybrid **B1** by chymotrypsin, trypsin, and pronase.

The stability of **B1** and **D2** was tested against trypsin and chymotrypsin—proteases of mammalian origin—and pronase, a mix of different enzymes extracted from bacterium *Streptomyces griseus*. **B1** and **D2** were incubated singularly in different vials with trypsin, chymotrypsin, and pronase at 37 °C (trypsin and pronase) and 30 °C (chymotrypsin), and the results were analyzed by comparing areas of the resulting RP-HPLC peaks after 6 and 24 h. The peptide-peptoid hybrid **B1** showed 6% degradation after 24 h of incubation with trypsin, 38% after pronase exposure, and no degradation after α-chymotrypsin exposure (Figure 3). Peptoid **D2** showed 0.2% degradation after 24 h incubation with pronase, and no degradation was observed by trypsin and chymotrypsin (see Supplementary Materials, Section S3).

### 2.3. Stability in Plasma

Plasma stability of **B1** and **D2** was tested as an estimation of their half-life in case of systemic absorption or intravenous administration. Plasma stability was determined by incubation of a known concentration of the peptoid/hybrid of interest with dog plasma up to 60 min at 37 °C and analyzing the concentration of the peptide in the supernatant by LC-MS/MS after protein precipitation. Results are presented as average of triplicates from a single donor. Three peptides with known stability profiles (Peptide 1–3) were used as controls in this study (see Table 1). **B1** showed some degradation in plasma, with a half-life of 28.9 minutes, while **D2** showed no detectable degradation and a half-life > 120 min (see Supplementary Materials, Section S6).

### 2.4. Stabilty in Liver Microsomes

Liver microsomal assay is used to determine hepatic intrinsic clearance of a drug and identification and profile of metabolites across species. Evaluation of microsomal stability of compounds in different species is common procedure in the drug discovery process to evaluate different metabolites in case of instability. In vitro stability of **B1** and **D2** in liver microsomes was investigated by incubating **B1** and **D2** with dog and human microsomes, and analyzing the resulting solution via LC-MS/MS to determine the residual amount of **B1** and **D2** after microsomal degradation. Both **B1** and **D2** were rather stable upon incubation with liver microsomes, both displaying in vitro half-lives >120 min (Table 1). After 60 min, 77% of D2 and 100% of B1 were recovered.

### 2.5. Plasma Protein Binding

The plasma protein binding properties of candidate drugs can influence their efficacy, safety, and distribution. The fraction of peptoid bound to proteins of plasma collected from dogs was determined using rapid equilibrium dialysis. Both **B1** and **D2** were highly bound (>98%) to dog plasma proteins (Table 2). A free fraction of 0.16% of the peptide-peptoid hybrid **B1** and 1.43% of the peptoid **D2** were detected via LC-MS/MS from dog plasma. Recoveries for both **B1** and **D2** were >70%.

### 2.6. In Vitro Release

**Figure 4:**
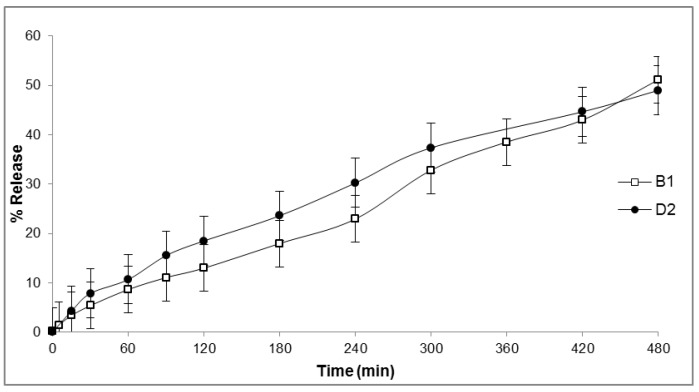
In vitro release of **B1** and **D2** from oil-in-water cream formulation. Mean ± SEM, *n* = 2.

To identify a suitable carrier for **B1** and **D2**, their release from three different formulations (oil-in-water cream, 20% poloxamer gel and ointment, see Table 3 and Section 4.6) was investigated. Release was evaluated as function of the amount of peptide diffused through a cellulose membrane in the receptor of a Franz cell and detected via RP-HPLC from 0 to 8 h. Approximately 50% of the content of both **B1** and **D2** was released from the oil-in-water cream over 8 h (Figure 4). The release kinetics indicated zero-order kinetics, and rates were comparable for the two compounds. On the contrary, no detectable release of **B1** and **D2** was observed from the 20% poloxamer gel and the ointment after eight hours (data not shown).

### 2.7. Skin Permeability

**Figure 5:**
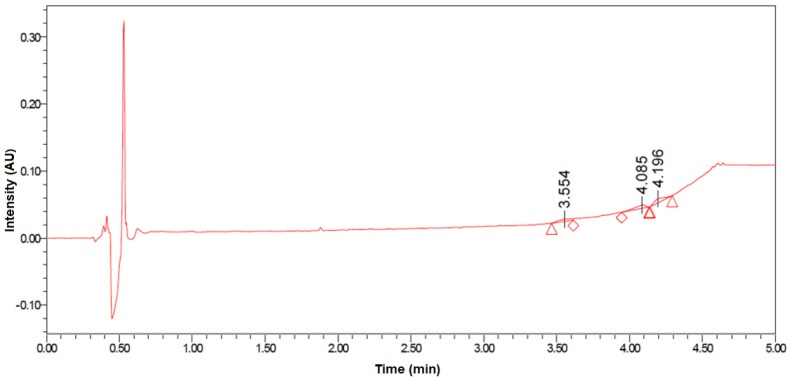
Skin penetration of B1 from 50% transcutol formulation detected via UV-UPLC after 24 h. No penetration of **B1** through dog skin is observed (UV-UPLC detection limit: 1 µg/L). Since **B1** and **D2** showed similar skin penetration pattern in all tested formulations, only representative data for **B1** are shown.

To evaluate the absorption of **B1** and **D2** into systemic circulation after topical administration, their in vitro permeation profile through excised dog skin was investigated in different excipient conditions, with and without the presence of a skin permeability modifier (transcutol). For each permeation study, donor chambers contained formulation at suitable concentration of peptoid for UPLC detection of penetration of 1% of total content and every sample was present three times. The experiment was repeated twice. Permeation was measured as a function of the concentration detected in the donor chamber of a Franz cell apparatus. A representative chromatogram is shown in Figure 5. No, or negligible, permeation (<1%) of **B1** and **D2** applied in any of the vehicles was detected after 48 h, suggesting practically no skin penetration of **B1** and **D2**. However, the presented results do not rule out potential partitioning into the stratum corneum.

## 3. Discussion

There is an urgent need for novel antibacterials specifically tailored for veterinary use only. Developing such agents for veterinary medicine would address the current lack of therapeutic options within this field, and reduce the use of critically important antibiotics approved for humans in the treatment of animal infections [19,20,21].

AMPs act quickly and selectively at micromolar concentrations, are rarely associated with antimicrobial resistance, and can be structurally optimized by chemical synthesis [22,23,24]. Their main limitations as therapeutics are their very short in vivo half-life and high production costs. Despite this, to date several AMPs are under commercial development [25]. De novo design of AMPs and peptidomimetics, such as peptoids [26,27,28,29,30], peptide-peptoid hybrids [31,32,33] and α-peptide/β-peptoids [34,35] have been successful strategies to increase the in vivo stability. The therapeutic potential of peptoids resides in their biological activity comparable to the parent peptide, and their resistance to proteolytic degradation [36]. Despite the large interest in developing peptoid-based antimicrobial agents, though, only a few studies on ADME properties of peptoid exist [37,38,39,40,41]. However, these are mainly limited to short peptoids—up to four residues. In this study, we describe a peptide-peptoid hybrid, **B1**, and a peptoid, **D2**, that were highly active against *S. pseudintermedius* (MIC 2–4 µg/mL). Moreover, the in vitro ADME properties of these peptoid-based antimicrobials were investigated in order to assess their potential as topical drug molecules to treat canine pyoderma.

Topical antimicrobial treatment of canine superficial pyoderma is favorable to systemic treatment, as very high concentrations can be reached at the site of infection. Furthermore, it minimizes the side effects and resistance development in other body sites, including the intestinal tract [6]. Creams and ointments are currently employed for the treatment of staphylococcal infections in veterinary medicine [42] and AMPs formulated in ointments (neomycin, bacitracin and polymyxin B [43]), cream (pexiganan) [44] and poloxamer gel (vancomycin) [45] are known.

To our knowledge there is no evidence in the literature of topical formulation studies of peptoid-based molecules, although they have been indicated as promising compounds for topical application [46]. To characterize the potential of **B1** and **D2** for topical treatment, we evaluated their release from three potential formulations with different hydrophilicity features (a hydrogel, an ointment and an oil-in-water cream), and assessed their permeation through canine skin. No release was observed when **B1** and **D2** were formulated in a poloxamer gel or an ointment. Only oil-in-water cream indicated suitability for topical delivery of **B1** and **D2**.

The permeation across excised skin was found to be negligible for both compounds as expected from their physicochemical properties. The skin permeability is often the most difficult challenge to overcome for potential topical drugs [47]. Drugs with a molecular weight >500 Dalton are likely to fail penetrating the SC, the external layer of the skin. Furthermore, the diffusion through skin of positively charged peptides may be prevented by association with negatively charged constituents in the skin [48]. To assess the permeation through skin, **B1** and **D2** were tested alone and in combination with chemical penetration enhancers (ethanol and transcutol) modifying physical properties of the SC [49,50]. Ethanol is widely used in topical applications and has been previously reported as a skin-penetrating agent for its effect of lipid/protein extraction from the SC and for its “push effect” due to evaporation [51]. Transcutol (diethylene glycol monoethylether) increases the solubility of drugs in the skin and is frequently used as a transdermal penetration enhancer in recent years [49,52]. Even in the presence of enhancers known to modify the physical properties of the SC [50], no permeation was observed. These results overall indicate that **B1** and **D2** are suitable for topical administration to skin. The lack of permeation likely predicts no systemic absorption of **B1** and **D2** following topical administration in vivo. Skin penetration of peptides is generally difficult: as an example, a recent paper reports of 5000 publications on formulation and clinical trials for the improvement of penetration of cyclosporine [53], a naturally-occurring multiple *N*-methylated cyclic peptide with a MW similar to **D2**. Further experiments are needed to evaluate the extent of the penetration of **B1** and **D2** into the skin layers, and in case of skin lesions caused by pyoderma, including in vivo studies. However, these initial results indicate that **B1** and **D2** are suitable for the treatment of superficial infections.

To characterize in vitro the behavior of **B1** and **D2** if systemically absorbed, their metabolic stability, hemolytic activity, and plasma protein binding were evaluated along with their susceptibility to proteolysis by trypsin, chymotrypsin and pronase. The peptide-peptoid hybrid **B1** only showed 6% degradation after 24 h of incubation with trypsin and 38% with pronase, while **D2** was stable. Our result is in good agreement with one of the earliest studies on stability of peptoids [54]. In that study, homologous l-amino acid, d-amino acid, and *N*-substituted glycine peptide and peptoid oligomers were incubated with different proteases from each major class (carboxypeptidase A, chymotrypsin, elastase, papain, pepsin, trypsin). The authors found that the analogues were virtually intact, while the l-peptides were rapidly degraded. 

In our study, the peptoid **D2** was also stable to plasma proteases, while the initial concentration of the peptide-peptoid hybrid **B1** was halved in about 30 min. Results indicate an increased stability in dog plasma conferred by the introduction of peptoid residues, while the presence of peptide bonds results in lower stability. Furthermore, the N-terminus of a peptide correlates with its resistance to proteolysis in plasma [15], and that may correspond to the decreased metabolic stability of the peptide-peptoid hybrid B1 as well. Although the cleavage site is not known with certainty, the presence of two *N*-terminal Lys residues suggests the site of proteolytic susceptibility. Stability to proteases is also relevant when considering topical applications, due to the presence of proteolytic enzymes of bacteria and skin. In one of the few studies addressing the in vivo pharmacokinetics of peptoids, [37] compared the distribution of a tripeptoid and a tetrapeptide after IV administration. The peptoid was not degraded and rapidly excreted, while the degradation products of the peptide were detected in peripheral body sites [37]. Similar results were reported by [41], who reported complete metabolic degradation of a labelled tripeptide in blood after two hours compared to stability of a labelled tripeptoid.

Both **B1** and **D2** showed good stability to in vitro degradation by liver microsomes, an assay which has been previously reported to correlate with in vivo data [55]. Half-lives for **B1** and **D2** were higher than 2 h, indicating a trend of low-medium clearance, compared to controls with known behavior. This suggests that **B1** and **D2** are not readily susceptible to degradation by liver metabolism. Furthermore, the skin contains metabolizing enzymes similar to the liver (CYP and UDP-glucuronosyltransferase) [56], hence, the assay may aid in predicting stability after cutaneous administration.

Toxicity is an important issue when developing antimicrobial agents. Hemolysis is a standard assay used when screening antimicrobial peptides. The concentration of **B1** resulting in 50% of human blood hemolysis was close to 256 μg/mL, comparable to approximately 100× MIC, while hemolysis of **D2** at the highest concentration tested did not reach 30%. A similar trend also appeared when the experiment was repeated with dog blood. This could be explained by the higher structural flexibility of peptoid chains compared to peptides and a different type interaction with erythrocyte membranes. The absence of backbone hydrogen bonds in peptoids prevents backbone-driven aggregation and makes peptoids better membrane permeating agents [57]. Compared to **B1**, the peptoid **D2** has a reduced number of hydrogen donors due to the peptoid backbone, higher hydrophilicity (featuring five positive charges, see Figure 1) and a higher degree of rotational flexibility. Rotational flexibility [58] is generally directly proportional to molecular weight, and it counts non-ring bonds to nonterminal non-hydrogen atoms. Despite the promising results, toxicity needs to be further assessed, e.g., by cytotoxicity to fibroblasts or other skin cells, as well as in vivo.

Both **B1** and **D2** have high protein binding, as both were >98% bound to plasma proteins in equilibrium dialysis in vitro. Plasma protein binding is also likely to occur with administration to a site of infection. Other antimicrobial peptides and drug candidates are also highly protein bound [59], especially apolipoprotein and serum albumin. Despite some inhibition of antimicrobial activity [60], these compounds retained part of their antimicrobial activity and, furthermore, were less cytotoxic at the site of action [61].

## 4. Materials and Methods

### 4.1. Synthesis and Characterization of Peptide-Peptoid Hybrid (**B1**) and Peptoid (**D2**)

HOAt (1-hydroxy-7-azabenzotriazole) and HATU (1-[Bis(dimethylamino)methylene]-1*H*-1,2,3-triazolo[4,5-b]pyridinium 3-oxid hexafluorophosphate) were from GL Biochem Shanghai (Shanghai, China). The compounds 1-naphthylmethylamine, 4-methylbenzylamine, butylamine, DIPCDI (*N*,*N*-diisopropylcarbodiimide), DIEA (diisopropylamine) and TIS (triisopropylsilane) were from Sigma-Aldrich (Copenhagen, Denmark. TentaGel SRAM (loading 0.2 mmol/g), TFA (trifluoroacetic acid), piperidine, and Fmoc-protected amino acids were purchased from Iris-Biotech GmbH (Marktredwitz, Germany). Disposable 5-mL polypropylene reactors fitted with a PTFE filter were acquired from Thermo Scientific (Hvidovre, Denmark).

Acetic acid and DMF (dimethylformamide), DCM (dichloromethane), ACN (acetonitrile) were purchased from VWR (Copenhagen, Denmark). All reagents and solvents were used without further purification. The manual solid-phase synthesis of peptide-peptoid hybrid **B1** and peptoid **D2** were carried out in disposable syringes on 100 mg of TentaGel S RAM (loading 0.2 mmol/g) using Fmoc chemistry for amino acids and the submonomer approach for peptoid building blocks as previously described [31]. Following synthesis, the product was cleaved from the resin using TFA/H_2_O/triisopopylsilane (95:2.5:2.5). Crude products were purified by preparative RP-HPLC until ≥97% purity was obtained (determined by analytical RP-HPLC (Waters Corporation, Milford, MA USA)). The identity of the products was verified by MALDI-TOF-MS (Bruker Daltonik GmbH, Bremen, Germany) (see supplementary information, Section S2).

### 4.2. Minimum Inhibitory Concentration

The antimicrobial activity of **B1** and **D2** was investigated by determining their minimum inhibitory concentration (MIC) against a methicillin-susceptible and a methicillin-resistant strain of *Staphylococcus pseudintermedius* isolated from canine skin and ears at the Veterinary Diagnostic laboratory at University of Copenhagen, Denmark. Assays were performed by microbroth dilution of test compounds in Mueller-Hinton broth (MHB) in microtiter plates (Corning 96-well clear polypropylene round bottom plates, non-treated) according to the Clinical and Laboratory Standard Institute guidelines [62]. The compounds were tested in concentrations ranging from 1 to 64 μg/mL (1, 2, 4, 8, 16, 32, 64 μg/mL). Briefly, bacteria were streaked on blood agar plates and incubated overnight at 37 °C. From these plates, colonies were suspended in saline solution and adjusted by nephelometer to obtain turbidity equivalent to a 0.5 McFarland standard. The suspension was diluted 1:100 in sterile 0.9% (*w*/*v*) saline, and 50 μL of this suspension was transferred to each well of the microtiter plate containing 50 μL of peptide solution in water, resulting in a concentration of 5 × 10^5^ CFU/well. The microtiter plate was sealed and incubated at 35 °C for 16–20 h. Results were read by visual inspection.

### 4.3. Hemolytic Activity

EC_10_ and EC_50_ values represent concentrations that result in 10 and 50% hemolysis, respectively. The EC_10_ and EC_50_ values were determined for **B1** and **D2**, as previously described [63]. Briefly, two-fold serial dilutions (1 to 256 μg/mL) were incubated in PBS with a suspension of freshly withdrawn human red blood cells in PBS in 96-well v-shaped microtiter plates at 37 °C for 1 h covered with Microseal foil to avoid evaporation, and transferred to 96-well flat bottomed microtiter plates. The release of hemoglobin was detected by measuring the absorbance at 414 nm by a UV spectrophotometer (VERSAmax™ Tunable Microplate Reader, Molecular Devices, San José, CA, USA). PBS and melittin were used as negative and positive controls, respectively. All human blood samples were obtained after informed consent of the voluntary donors upon understanding of the research purposes, within local hospitals and according to local guidelines.

### 4.4. Stability of Peptide-Peptoid Hybrid (**B1**) and Peptoid (**D2**)

#### 4.4.1. Stability to Proteases

The effect of proteases on **B1 and D2** was tested by incubation of the compounds at 37 °C with three commercially-available proteases (trypsin and α-chymotrypsin from bovine pancreas, and pronase from *Streptomyces griseus*, all purchased from Sigma Aldrich). Solutions, concentrations, and reaction temperatures were selected according to manufacturer’s recommendations for total peptide digestion. A stock solution of 1 mM trypsin was prepared in 1 mM Tris HCl buffer solution containing 20 mM Ca^2+^. For the proteolytic stability assay, a 10 µg/mL trypsin concentration and 1 mg/mL concentration of **B1 and D2** in 0.1 M Tris buffer were used to obtain a 1:100 enzyme:peptide ratio. Reaction was conducted at 37 °C for 6 h. Chymotrypsin was solubilized at 10 mg/mL in 1 mM HCl solution containing 2 mM CaCl_2_. A ratio of 1:60 chymotrypsin: peptide was used (17 µg/mL chymotrypsin, 1 mg/mL peptide). Chymotrypsin reaction was conducted in 100 mM Tris HCl containing 10 mM CaCl_2_, pH 7.8 at 30 °C. The reaction with pronase was conducted, following the enzyme producer’s recommendation, using 0.2 µM **B1** and **D2** and 1% pronase powder (*w*/*w*) in 50 mM ammonium bicarbonate buffer at pH 8 at 37 °C. At time 0, 3, and 6 h, 50 µL aliquots were removed for RP-HPLC analysis (see supplementary Materials, Section S3).

#### 4.4.2. Stability in Plasma

Dog plasma was collected from a single Beagle dog at the research facilities of Zoetis Animal Health (Kalamazoo, MI, USA) and frozen immediately until use. Proprietary linear peptides with a short and medium half-life and a cyclic peptide with a long half-life were used as controls (Table 2). **B1**, **D2**, and controls were diluted to 10 μM in 2% (*w*/*v*) BSA containing 0.2% (*v*/*v*) formic acid to prevent non-specific binding to materials. Test compounds were added to plasma diluted 1:1 with PBS buffer pH 7.4 and preheated for 5 min at 37 °C. Samples were removed at 0, 5, 10, 20, 30, and 60 min. The reaction was stopped by protein precipitation by adding a 9:1 (*v*/*v*) ACN:MeOH mixture followed by centrifugation at 10,000 rpm for 5 min, and the remaining amount of intact test compounds in the supernatant was quantified via API Sciex 4000Q LC-MS/MS (Framingham, MA, USA) (coupled with an HPLC column to filter off the plasma proteins—see Supplementary Materials, Section S6) upon completion of the assay. Experiments were performed in quadruplicate. The half-life values were calculated on the basis of the rate of substrate disappearance (Equation (1)) and calculating the in vitro half-lives (t½) (Equation (2)):

% compound remaining = Ae^−kt^, (1)

where k is the rate constant for disappearance of parent compound, t is time and A is the starting concentration and is close to 100 when data is normalized to the zero time point:

t_1/2_ = 0.693/k, (2)

#### 4.4.3. Stability to Liver Microsomes

Evaluation of stability of **B1** and **D2** to the enzymes in liver microsomes was performed with a robotic system for liquid handling (Hamilton Microlab STAR, Hamilton Robotics, Reno, NV, USA). Dog and human microsomes were obtained from XenoTech LLC (Kansas City, KS, USA) and reconstituted in buffer containing d-(l)-isocitrate trisodium salt and 10 U/mL isocitrate dehydrogenase type IV glycerol solution (procine). A stock solution of 10 mM substrate (**B1**, **D2** or controls, proprietary peptides of Zoetis, Kalamazoo, MI, USA) was diluted 1:1000 in 1% (*v*/*v*) ACN before adding to the reaction mixture. The reaction was initiated by adding NADPH and substrate to reconstituted microsomes. A final concentration of 1 µM substrate was incubated with 0.5 mg/mL microsomes in the presence of 1 mM NADP^+^ in 100 mM PBS with 1 mM MgCl_2_. Samples were collected at 0, 5, 10, 20, 30, and 60 min. The reaction was conducted at 37 °C before termination by addition of ACN, and analysis was conducted by LC-MS/MS (similar to the method described above). Controls with no drug and with no microsome regeneration system (buffer only) were included. All samples were prepared in triplicate. The rate of degradation by metabolism was measured on the basis of the difference of substrate concentration remaining at every time point. Metabolic half-lives were calculated as mentioned above.

### 4.5. Plasma Protein Binding

The in vitro plasma protein binding properties of **B1** and **D2** were investigated with the use of Thermo Scientific™ rapid equilibrium dialysis polypropylene 96-well plates. Plate inserts comprised two side-by-side chambers separated by a dialysis membrane (8K MWCO). The chambers contained 1100 µL plasma or 350 µL dialysis buffer (PBS buffer pH 7.4), respectively. Dogplasma lot was identical to the one used in the plasma stability assay. Ten mM stock solutions of **B1** and **D2** were diluted 1:50 in water, and 200 µL of diluted **B1**, **D2**, and control compound (sarolaner, >99% protein bound) were added to the wells containing plasma in an automated robot for liquid handling. Plates were sealed and incubated at 37 °C for 4 h on a shaker. Each test was run in quadruplicate in dog plasma. Upon completion of the assay, the plasma protein binding was determined by protein precipitation with ACN of both plasma and PBS, and drug levels were quantified via LC-MS/MS as described above. The percentage of bound compounds was determined from peak areas using Equations (3) and (4):

% free = (concentration buffer chamber/concentration plasma chamber) × 100%(3)

% bound = 100% − % free(4)

### 4.6. Formulation Preparation

An oil-in-water cream and an ointment were prepared as described by Danish Health and Medicines Authority [64]. For the oil-in-water cream, ingredients of the oil phase (Table 3) were melted on a water bath at 70 °C and mixed with the water phase, which was previously brought to boil and cooled down to 65–70 °C. The mixture was stirred until cooled. The ointment formulation was prepared by mixing melted Vaseline (80%) with paraffin oil (20%) on a water bath and allowed to cool under stirring. The cold method was used for preparation of the 20% (*w*/*w*) poloxamer gel [65], where the poloxamer powder was dissolved in purified water at 4 °C and subsequently left overnight at 2–8 °C to stabilize. All formulations were stored at 4 °C for maximum seven days before use. Low doses of **B1** or **D2** were loaded into the formulations just before initializing the release experiment by first dissolving an appropriate amount of compound in 40 to 60 µL of water (or 96% ethanol in case of the ointment) and mixing this into 1 mL of the formulation to obtain a final concentration of 600 µg/mL. For the hydrogel, the compounds dissolved in water were mixed with the cold poloxamer solution before gelling at room temperature.

### 4.7. In Vitro Drug Release

The release of **B1** and **D2** from the formulations was determined by utilizing the Franz cell apparatus. Approximately 200 µL of each formulation was filled into the donor chambers of the Franz cells via a 1 mL syringe ensuring contact with the cellulose membrane separating the donor compartment from the receptor compartment. The donor compartment was maintained at room temperature (25 ± 1 °C) and the membrane diffusion area was 1.57 cm^2^. Activated cellulose membranes (washed 3× with cold water and 3× with 100 °C warm water under stirring for 5 min) were used. The receptor medium (3 mL PBS, pH 7.4) was stirred using magnetic stirring and thermo-regulated to 36 °C by a water jacket. Samples (200 μL) were collected from the receptor compartment through the sampling port over 8 h, and the withdrawn volume was replaced with an equal volume of buffer at every time point. The samples were analyzed by a Waters 600E RP-HPLC equipped with Empower 3 software (Waters Corporation, Milford, MA, USA): 30 min run time with 20 min linear gradient from 0.1% (*v*/*v*) TFA in water to 30% (*v*/*v*) ACN:H_2_O with 0.1% (*v*/*v*) TFA (90:10). The amounts of **B1** and **D2** released and diffused through the cellulose membrane were determined using the calibration curve as previously described. Experiments were performed in duplicate.

### 4.8. Skin Permeation

The amount of **B1** and **D2** that permeated excised dog skin using three different vehicles was determined ex vivo on an automated Franz cell apparatus. **B1** and **D2** were dissolved (10 mg/mL) in 20 mM sodium acetate buffer pH 5, in 50% (*v*/*v*) ethanol and in 50% (*v*/*v*) diethylene glycol monoethyl ether (Transcutol^®^) in sodium acetate buffer. Skin obtained from the dorsal side of Beagle dogs was pre-treated and stored at −20 °C until use. Pre-treated skin was partially shaved and consisted of stratum corneum (SC) and epidermis. On the day of the experiment, the skin was thawed at room temperature (RT) for one hour, cut in circles (diameter ≈ 2 cm), and stored at RT for 1 h prior to experiments. A circulating water bath was used to keep the cells at a temperature of 37 °C. Polysorbate 80 was used in the receptor chambers (containing 10 mL) to avoid non-specific binding of **B1** and **D2** to the glass. Prior to the experiment, the receptor chamber solution was degassed for 30 min at RT. Cells were primed and rinsed three times with 10 mL water and three times with 10 mL polysorbate 80 solution. The skin was then mounted on the diffusion cells with the SC facing upwards towards the donor compartment. Transepidermal water loss (TEWL) was measured for each piece of skin independently at the start of the experiment by utilizing Delfin VapoMeter in contact for 10 s with the skin mounted on the chamber. The receptor chambers were subsequently filled with further 10 mL polysorbate 80 solution and left to equilibrate for 150 min. Donor chambers contained 100 µL of formulation added in two steps (2 × 50 µL). Permeation experiments were conducted for 48 h, and donor chambers containing Transcutol^®^ were covered with tin foil to avoid any light exposure. At predetermined time points (2, 6, 12, 24, and 48 h), 1 mL of the receptor phase was withdrawn and replaced with the same amount of receptor medium. The amount of **B1** and **D2** in the withdrawn samples was determined by UPLC analysis. The experiment was conducted in duplicate.

## 5. Conclusions

In conclusion, the novel antimicrobial peptide-peptoid hybrid **B1** and peptoid **D2** displayed properties suitable for the topical treatment of *S. pseudintermedius* infections in, e.g., an oil-in-water cream and metabolic stability. The positively-charged peptoid-based molecules were found to not penetrate skin, thus, rendering them suitable for topical application when no systemic absorption is desired (e.g., to treat superficial pyoderma). Although in vitro results need to be confirmed by in vivo data, our results show how the presence of peptoid residues correlates with increased stability at conditions resembling in vivo metabolism and possibly with a decreased degree of hemolysis. To the authors’ knowledge, this study represents the first characterization of in vitro early ADME properties of peptoid-based antimicrobials.

## Figures and Tables

**Table 1:** Half-lives (minutes) of **B1** and **D2** and control peptides ^1^ in plasma and liver microsomes.

Compound	Plasma (Dog)	Liver Microsomes (Dog)	Liver Microsomes (Human)
**B1**	28.9	>120	>120
**D2**	>120	>120	>120
Peptide-1	>120	-	-
Peptide-2	<3	-	-
Peptide-3	81.2	-	-

^1^ Peptide-1, -2, and -3 are control peptides with long, medium and short plasma half-life, respectively.

**Table 2:** Protein binding of **B1**, **D2**, and control peptide after 4 h incubation in plasma.

Entry	% Average Recovery	SD ^1^	% Unbound	Bound	SD ^1^
**B1**	75.6	20.7	0.16	99.8	0.000
**D2**	70.8	6.5	1.43	98.6	0.005
Control	67.1	12.6	0.27	99.7	0.001

^1^ Mean ± SD (*n* = 4).

**Table 3:** Composition of the tested compounds.

Formulation	Ingredients	% (*w*/*w*)
Oil-in-water cream	Oil Phase	
	Polysorbate 80	0.5
	Cetostearyl alcohol	5
	Paraffin oil	5
	Glycerol monostearate	6
	Water Phase	
	Methyl parahydroxy-benzoate	0.1
	85% glycerol	4
	Sorbitol	7
	Purified water	72.4
Oinment		
	Paraffin oil	20
	Vaselin	80
Hydrogel		
	Poloxamer 407	20
	Purified water	80

## Supplementary Materials

Supplementary materials are available on line. Table S1: Gradient elution used for purification of compounds on preparative RP-HPLC. Table S2: Gradient elution used for analytical RP-HPLC , Table S3: Retention times, peak areas and relative peak areas of D2 as detected by RP-HPLC., Table S4: Final gradient elution UV-UPLC method used for the LC-MS/MS experiments., Table S5: Half-lives of peptide-peptoid hybrid B1 and peptoid D2 measured after 60 minutes of incubation at 37°C with dog plasma; Figure S1: Analytical HPLC chromatogram of compound B1, Figure S2: MALDI-TOF MS spectrum of B1, Figure S3: Analytical HPLC chromatogram of compound D2, Figure S4: MALDI-TOF MS spectrum of D2, Figure S5: Stability of D2 to pronase E, Figure S6: B1 in 50% Transcutol in acetate buffer pH 5 detected via UV-UPLC (day 0), Figure S7: B1 in 50% Transcutol in acetate buffer pH 5 detected via UV-UPLC (Day 1), Figure S8: Concentration-time profile in plasma of the compounds B1 and D2.
