# Supplementary material for: In Vitro ADME Properties of Two Novel Antimicrobial Peptoid-Based Compounds as Potential Agents against Canine Pyoderma
Source: Molecules. 2018 Mar 10;23(3):630. doi: 10.3390/molecules23030630 (PMC6017477; doi:10.3390/molecules23030630)
Supplement: Supplementary file 1 [file molecules-23-00630-s001.pdf]

Supplementary Materials

In Vitro ADME Properties of Two Novel Antimicrobial Peptoid-Based Compounds as Potential Agents against Canine Pyoderma

Ines Greco* [1,2,3], Bernard D. Hummel [3] , Jaspreet Vasir[3], Jeffrey L. Watts[3], Jason Koch[3] , Johannes E. Hansen [1], Hanne Mørck Nielsen[4], Peter Damborg[5], and Paul R. Hansen[1]*

[1] University of Copenhagen, Department of Drug Design and Pharmacology, Universitetsparken 2, 2100 Copenhagen Ø, Denmark; ines.greco@food.ku.dk (I.G.); rwn546@alumni.ku.dk (J.E.H.); prh@sund.ku.dk (P.R.H.)
[2] Present address: University of Copenhagen, Department of Food Science, Rolighedsvej 30, DK-1958 Frederiksberg, Denmark.
[3] Zoetis, Inc., 333 Portage St., 49007 Kalamazoo (MI), USA; bernard.d.hummel@zoetis.com (B.D.H).; jaspreet.vasir@zoetis.com (J.V.); jeffrey.l.watts@zoetis.com (J.L.W.); jason.koch2@zoetis.com (J.K.).
[4] University of Copenhagen, Department of Pharmacy, Universitetsparken 2, 2100 Copenhagen Ø, Denmark; hanne.morck@sund.ku.dk (H.M.N.)
[5] University of Copenhagen, Department of Veterinary and Animal Sciences, Stigbøjlen 4, 1870 Frederiksberg C, Denmark.; pedam@sund.ku.dk

Content:

S1: Preparative and Analytical RP-HPLC
S2: Analytical RP-HPLC chromatograms and MALDI-TOF MS spectra
S3: Degradation profile of D2 with pronase
S4: Stability of B1 in 50% transcutol in acetate buffer
S5: LC-MS/MS method
S6: Plasma stability of B1 and D2

S1: Preparative and Analytical RP-HPLC and MALDI-TOF mass spectrometry

S1.1 Purification by preparative HPLC

Crude products were dissolved in ACN (150 μL) and Milli-Q water (150 μL) and injected into a preparative RP-HPLC column (Waters XBridge™ Prep BEH C18 Column, 5 μm, 10 X 250 mm) equipped with a guard column (Waters Cartridge Holder PKG 10 x 10 mm). The system consisted of Waters In-Line Degasser, 600 Controller, 996 Photo Array Detector and 600 Pump. The purification was performed by gradient elution (Table S1) using the same mobile phases as for analytical RP-HPLC.

Table S1. Gradient elution used for purification of compounds on preparative RP-HPLC. % A and % B indicates the percentage of the mobile phases mixed at the given time.

Time (min)	Flow (mL/min)	% A	% B
	4	100	0
1	6	100	0
3	8.5	100	0
5	8.5	90	10
20	8.5	45	55

26	9	0	100
27	9	0	100
30	9	100	0
39	9	100	0
40	1.5	100	0

All compounds were purified by a 40 min run. Peaks were detected at 220 nm and 280 nm to separate impurities from the aromatic compounds.

The collected fractions were evaporated in vacuo and the dry product was transferred to a clean cryotube and freeze-dried overnight.

S1.2 Characterization by analytical HPLC

Purity was assessed by analytical RP-HPLC column (Waters XBridge™ BEH C18 Column, 130Å, 3.5 µm, 4.6 mm X 150 mm) The system consisted of Waters In-Line Degasser, 600 Controller, 2996 Photodiode Array Detector, 600 Pump and 717plus Autosampler.

Compounds were dissolved in H_2O:ACN:TFA (90:10:0.1) (1.5 mL). This solution (30 µL) and ACN (80 µL) were transferred into a HPLC vial. A volume of 10-15 µL was injected into the column and analysed in a 30 min run with 9 min delay until next injection. The analysis was performed by gradient elution (Table S2) using mobile phases: A: 0.01 % TFA in Milli-Q water and B: 90 % ACN in Milli-Q water + 0.01 %. During the delay 100% A was used with a flow of 1.5 mL/min. Peaks were detected at 220 nm. The ApexTrack™ algorithm was used for peak integration.

The same analytical method was used for evaluation of stability to proteases.

Table S2. Gradient elution used for analytical RP-HPLC . %A and %B indicate the percentage of the mobile phases mixed at the given time.

Time (min)	Flow (mL/min)	% A	% B
	**1.5**	**100**	**0**
**20**	**1.5**	**30**	**70**
**22**	**1.5**	**0**	**100**
**27**	**1.5**	**0**	**100**

**29**	**1.5**	**100**	**0**

S1.3 Characterization of B1 and D2 by MALDI-TOF mass spectrometry

Verification of molecular mass was achieved by MALDI-TOF MS. A droplet (0.5 μL) of a preparative HPLC fraction of the pure compound was applied on the target plate, and allowed to dry completely. Alternatively, a small amount of the dry compound was dissolved in Milli-Q water (700 μL) and ACN (300 μL) reaching a concentration in the range of 0.01-0.1 mg/mL. When dry, a matrix solution (0.5 μL) was added on top of the sample spot and allowed to dry completely. The matrix was prepared by dissolving alpha-cyano-4-hydroxycinnamic acid (10 mg) in ACN:H_2O:TFA (500:475:25) (1 mL).The molecular mass was analysed by a Bruker Microflex MALDI-TOF MS using the software FlexControl. Data was processed by software Flex Analysis.

S2 Analytical RP-HPLC chromatograms and MALDI-TOF MS spectra

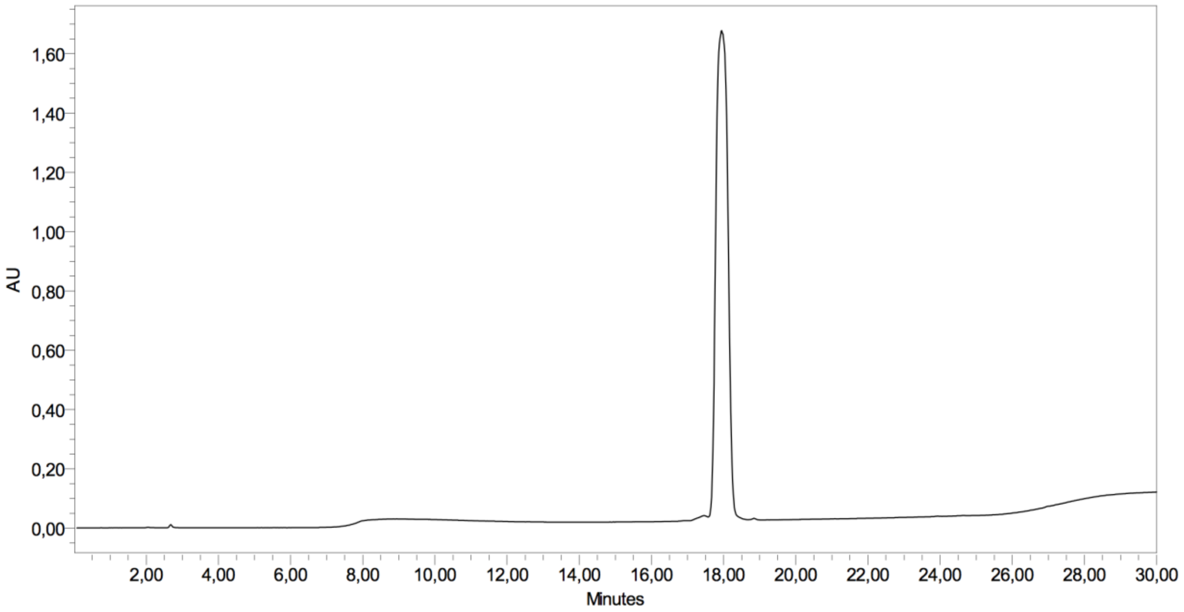

Figure S1 Analytical HPLC chromatogram of compound B1

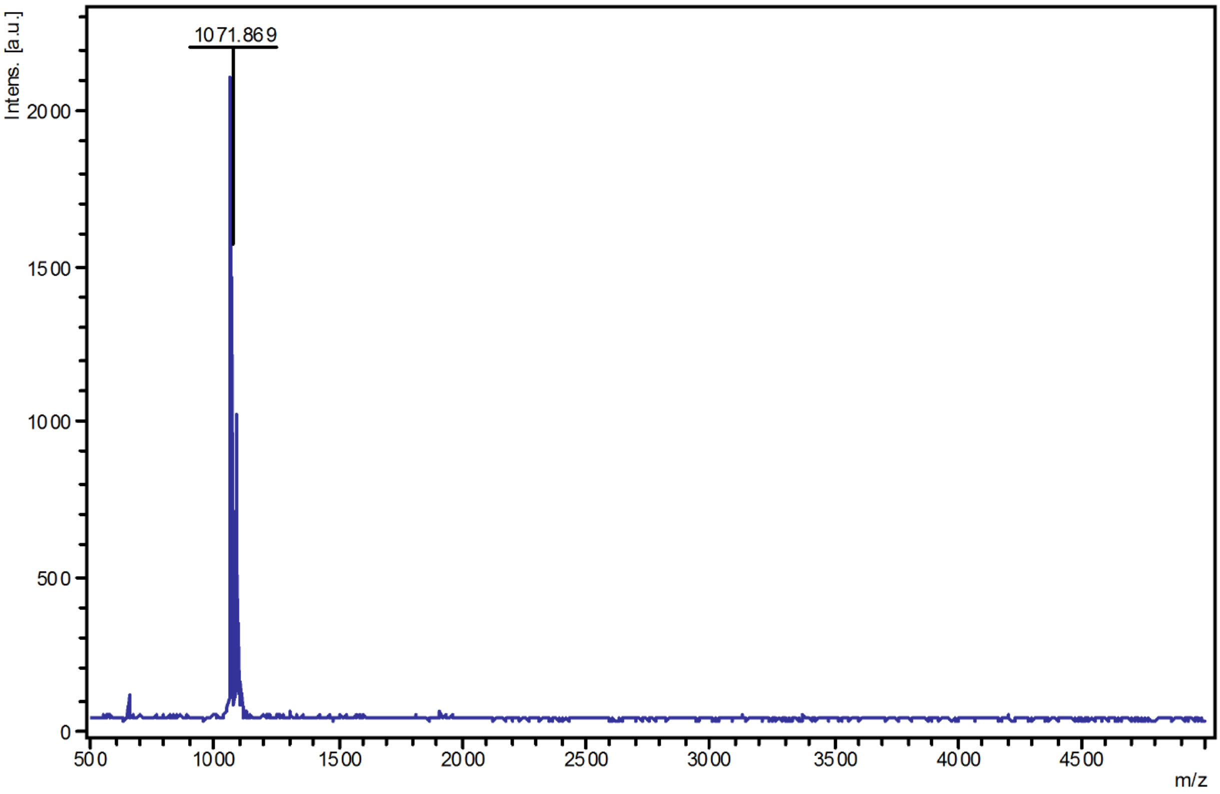

Figure S2 MALDI-TOF MS spectrum of B1 (calculated: 1070.39/observed (MH+): 1071.869)

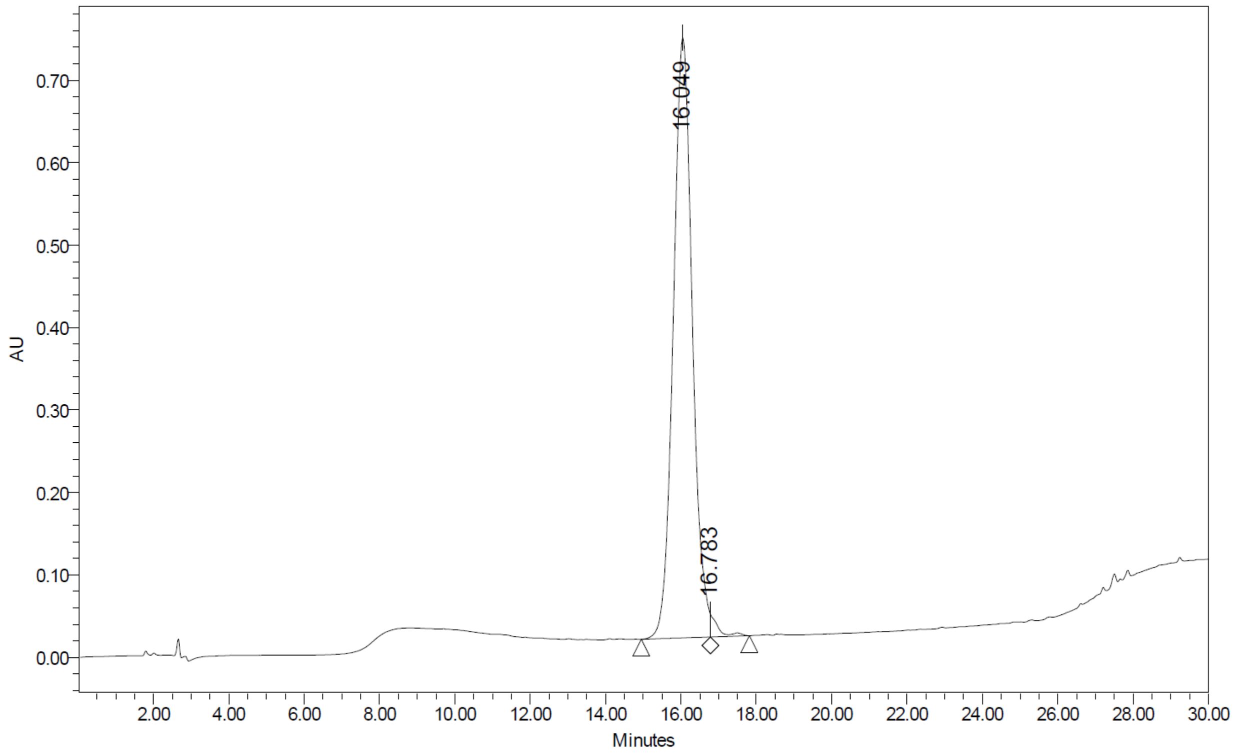

Figure 3 Analytical HPLC chromatogram of compound D2

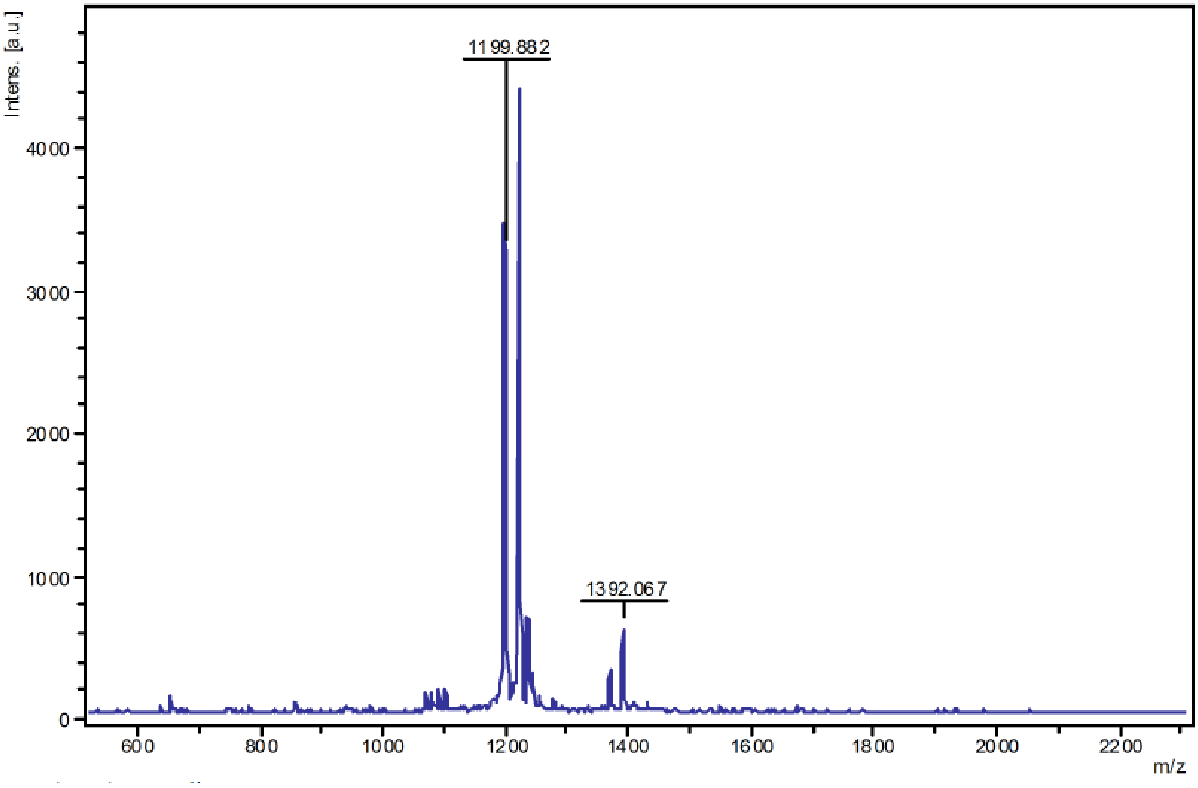

Figure S4 MALDI-TOF MS spectrum of D2 (calculated: 1199.53/observed (MH+): 1199.882). Sodium adduct at

1220.695.

S3 Degradation profile of D2 with pronase after 24 hours

D2 shows stability when exposed to the mix of proteases of bacterial origin (pronase from *S. griseus*).

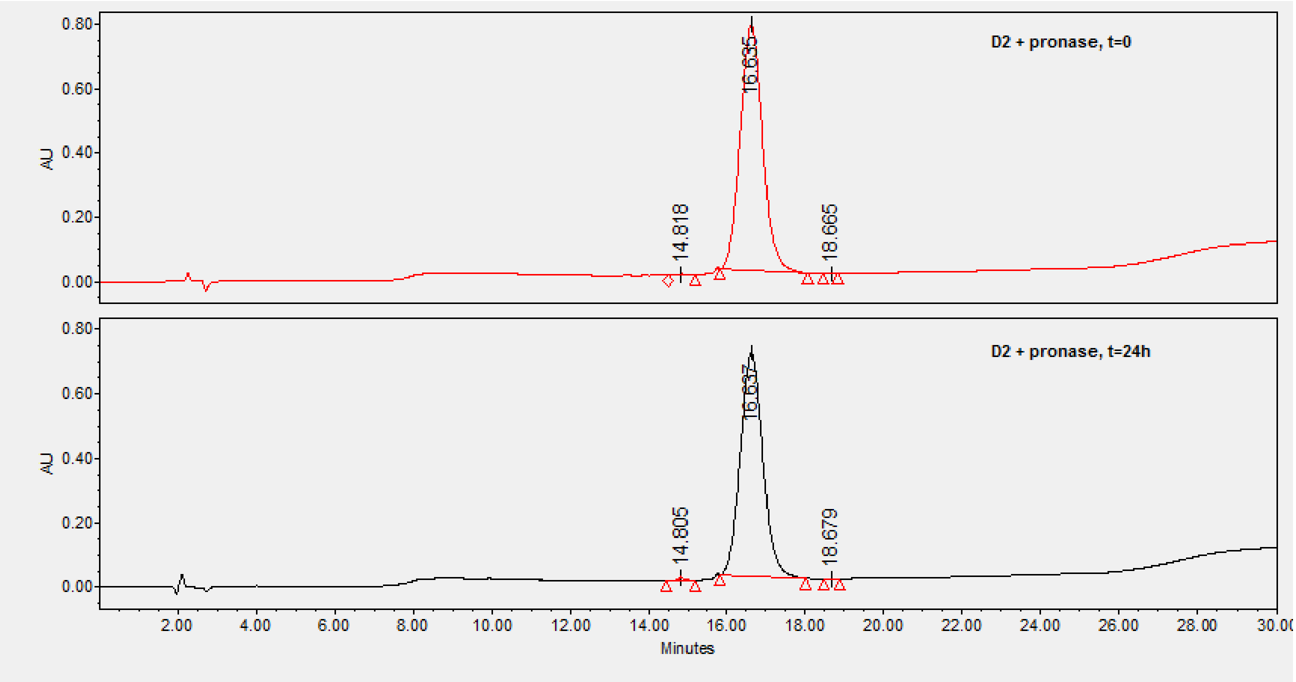

Figure S5 Stability of D2 to pronase E. Chromatograms at time 0 (top) and 24 hours (bottom) are shown. Degradation profile detected via RP-HPLC.

Table S3. Retention times, peak areas and relative peak areas (indicated as percentage) of D2 as detected by RP-HPLC at 0 (a) and 24 hours (b)

a)

	t=0	Retention Time	Area	% Area
2		16.635	33282781	99.79
1		14.819	69939	0.21

b)

	Name	Retention Time	Area	% Area
1		14.805	135287	0.44
2		16.636	30303329	99.56

S4 B1 is stable in 50% transcutol in acetate buffer (detected by UV-UPLC)

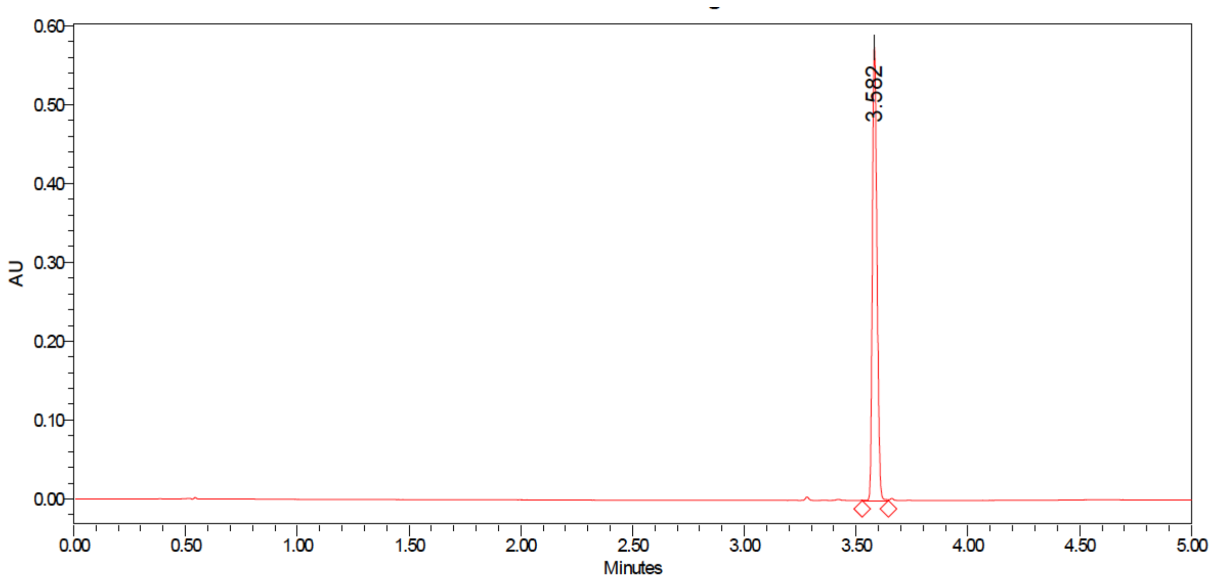

Figure S6 B1 in 50% Transcutol in acetate buffer pH 5 detected via UV-UPLC (Day 0).

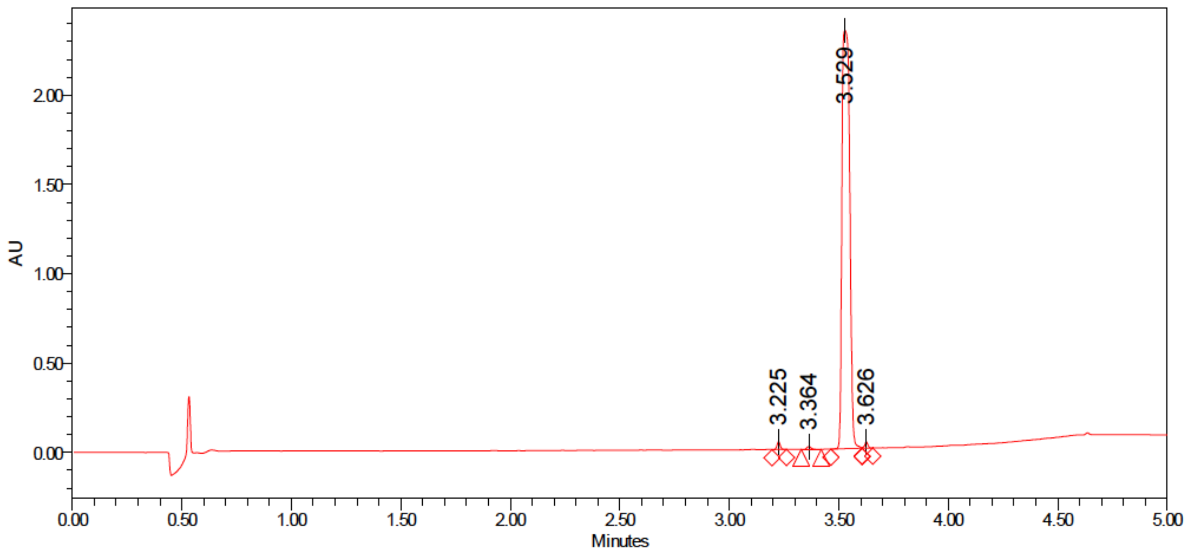

Figure S7 B1 in 50% Transcutol in acetate buffer pH 5 detected via UV-UPLC (Day 1).

S5 LC-MS/MS method

S5.1 UV-UPLC method

5-60% ACN in 2 minutes, 0.1% Formic Acid

(Waters Acquity UV-UPLC, C18 column, 2.1x54 mm, T=60°C, equilibration time= 0.1 min)

Table S4 Final gradient elution UV-UPLC method used for the LC-MS/MS experiments. %A and %B indicate the percentage of the mobile phases mixed at the given time.

Time (min)	Flow (ml/min)	%A	%B
**0**	**0.6**	**95**	**5**
**0.4**	**0.6**	**95**	**5**
**2.4**	**0.6**	**40**	**60**
**3**	**0.6**	**20**	**80**
**4**	**0.6**	**20**	**80**
**4.4**	**0.6**	**95**	**5**
**5**	**0.6**	**95**	**5**

A= 0.1% Formic Acid in H_2O
B= 0.1% Formic Acid in ACN

S5.2 MS/MS method

Instrument: Sciex API 4000 QTRAP LC-MS/MS

Software: Analyst 1.5.2
Acquisition duration: 3 minutes
Scan type: MRM
Polarity: Positive
Ion Source: Turbo Spray
Resolution Q1: Unit
Resolution Q3: Unit

D2: Q1 mass (Da) 400.7; Q3 mass (Da) 348.1 and 141.1
B1: Q1 mass (Da) 357.8; Q3 mass (Da) 466.0 and 141.1

S6 Plasma Stability of B1 and D2

Table S5 Half-lives of peptide-peptoid hybrid B1 and peptoid D2 measured after 60 minutes of incubation at 37°C with dog plasma.

Compound	Half-life (min)	Species
B1	28.9	Dog
D2	>120	Dog

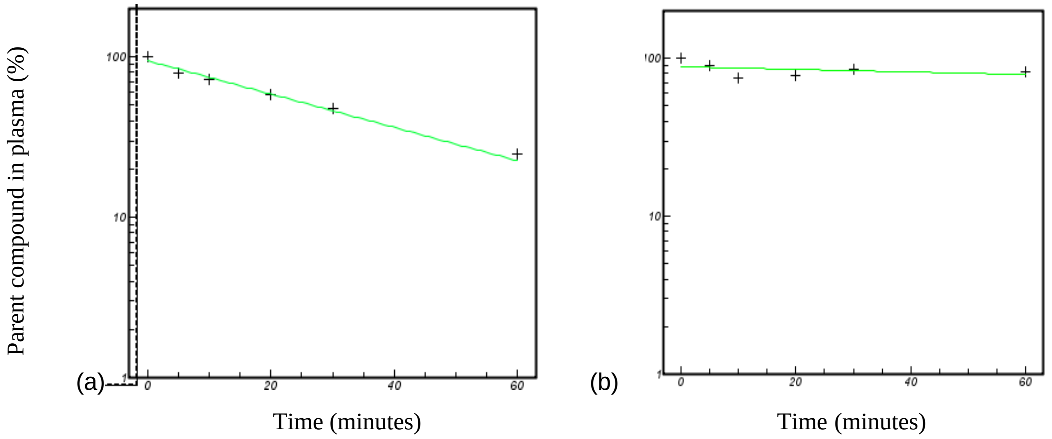

Figure S8 Concentration-time profile in plasma of the compounds B1 (a) and D2 (b).
